# Elevated Mitochondrial DNA Copy Number in Peripheral Blood and Tissue Predict the Opposite Outcome of Cancer: A Meta-Analysis

**DOI:** 10.1038/srep37404

**Published:** 2016-11-18

**Authors:** Nan Chen, Shu Wen, Xiaoru Sun, Qian Fang, Lin Huang, Shuai Liu, Wanling Li, Meng Qiu

**Affiliations:** 1 West China School of Medicine/West China Hospital, Sichuan University, Chengdu 610041, China; 2 Department of Medical Oncology, Cancer Center, the State Key Laboratory of Biotherapy, West China Hospital, Sichuan University, No. 37, Guoxue Alley, Chengdu, Sichuan, 610041, China

## Abstract

Previous studies have suggested that mitochondrial DNA (mtDNA) copy number was associated with cancer risk. However, no solid conclusion revealed the potential predictive value of mtDNA copy number for cancer prognosis. The present meta-analysis was performed to clarify the problem. Hence, we performed a systematic search in PubMed, EmBase, Web of Science databases independently and a total of eighteen studies comprising 3961 cases satisfied the criteria and finally enrolled. Our results didn’t show the association between them but significant heterogeneity in overall analysis (OS: HR = 0.923, 95% CI: 0.653–1.306, p = 0.652; DFS: HR = 0.997, 95% CI: 0.599–1.659, p = 0.99). However, subgroup analysis stratified by sample came to the opposite conclusion. High level mitochondrial DNA copy number in peripheral blood predicted a poor cancer prognosis (OS: HR = 1.624, 95% CI: 1.211–2.177, p = 0.001; DFS: HR = 1.582, 95% CI: 1.026–2.439, p = 0.038) while patients with high level mitochondrial DNA copy number in tumor tissue exhibited better outcomes (OS: HR = 0.604 95% CI: 0.406–0.899, p = 0.013; DFS: HR = 0.593, 95% CI: 0.411–0.857, p = 0.005). These findings were further proved in detailed analyses in blood or tissue subgroup. In conclusion, our study suggested the elevated mtDNA copy number in peripheral blood predicted a poor cancer prognosis while the better outcome was presented among patients with elevated mtDNA copy number in tumor tissue.

Cancer is one of the leading causes of mortality all over the world^1^. Although significant achievements have been made in the area of cancer therapy, the occurrence of cancer was still on the increase especially in developing countries, and the mortality rates remained high globally^1^. Based on the latest GLOBOCAN report, there were 14.1 million new cancer cases and 8.2 million deaths annually worldwide^1^. At present, identification of the specific predictive or prognostic biomarkers have been explored in various cancers. For example, the over-expression of SIRT1 may predict a worse outcome in colorectal carcinoma^2^. However, the promising biomarkers which contributed effective role in the prognosis of cancer have been in the balance and warranted for further investigation.

Mitochondria functions in a range of bioactivities like cellular energy metabolism, reactive oxygen species induction, and apoptosis through ATP production and metabolites generation in the tricarboxylic acid cycle, also involved in the mitochondria-to-nucleus signaling pathway to the regulation of mitochondrial morphology, activity and function^3^,^4^. Compared to nuclear DNA, mitochondrial DNA (mtDNA) was more susceptible to external or internal factors due to the characteristics of mtDNA including intron-less, lack of histones, inefficient mtDNA proof-reading and mtDNA repair system^5^. Since Warburg observed an increased capacity of glycolysis in cancerous cells contrasting to normal cells and thus supposed altered mitochondrial dysfunction contributed to cancer, subsequent studies revealed the main driver of tumorigenesis was an insufficient cellular respiration caused by mitochondrial dysfunction^6^,^7^. As mitochondria might playing a crucial role in cancer susceptibility and development^8^, it is reasonable to speculate that mtDNA variations or alteration of mtDNA copy number may be closely related to various cancers.

mtDNA copy number fluctuated from 10^2^ to 10^4^ copies per cell varying in response to the physiological environment surrounding the cell and it was mainly regulated by mitochondrial transcription factor A (TFAM) mtDNA polymerase γ^9^,^10^. With the rapid advance of molecular biological technique, a series of new technical methods makes it impossible to estimate mtDNA copy number accurately and the magnitude of quantitative changes in mtDNA copy number have been observed in many types of malignancies, such as gastric cancer, head and neck cancer and colorectal cancer (CRC), etc^11^,^12^,^13^,^14^. The mtDNA copy number has been regarded as a hot spot in the field of cancer research process. A recent systematic review suggested that elevated mtDNA copy number was associated with a higher risk for lymphoma, but a lower risk for skeleton cancer through the comprehensive assessment of pooled studies^15^. Much more attention has been paid to independently evaluate the prognostic value of mtDNA copy number in various cancer types, and the results are controversial, even in the studies on the same type of cancer like colorectal cancer^16^,^17^,^18^. In general, the existing research is focused on the specific cancers types and there are few parallels on experimental methods quantifying mtDNA copy number between these studies. Despite bearing the certain differences, these recordings have so far not been systematically analyzed and thus the association between mtDNA copy number and clinical cancer prognosis remains unclear.

Therefore, it was timely and necessary to analyze globally the prognostic value of mtDNA copy number in larger population to fill the gap of lacking the related comprehensive analysis and clarify the pending issue. In this article, we performed a meta-analysis integrating the valid results from conditional homogeneous studies to quantitatively review the effect of high versus low mtDNA copy number on the survival of patients in peripheral blood and tissue with some specific types of cancer, respectively. We also compared the predicting value of mtDNA in terms of origin of population and other characteristics through subgroup analysis.

## Results

### Characteristics of included studies

**Figure 1:**
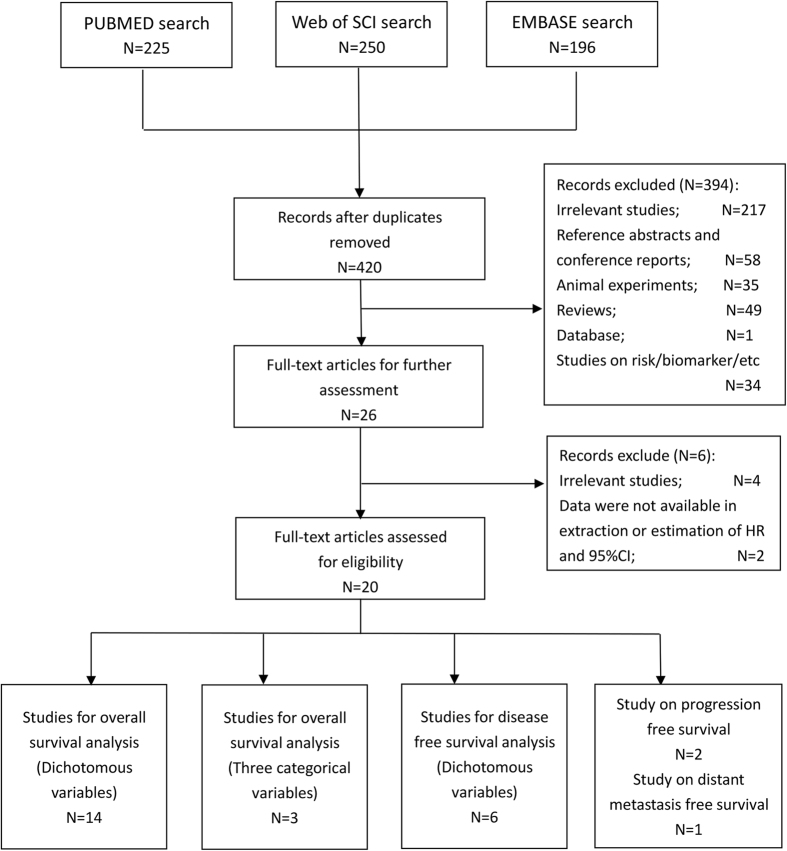
Flow chart of the literature selection.

A total of 420 studies were incorporated into our primary filtration after primary systemic search in main databases mentioned above (Fig. 1). After a thorough review of titles and abstracts by two investigators independently, 394 studies were excluded and the full texts of remaining 26 references have also been artificial retrieved for further identification. Finally a total of 20 retrospective studies comprising 5413 patients were included in our study and 18 studies were used for analysis^16^,^17^,^18^,^19^,^20^,^21^,^22^,^25^,^26^,^27^,^28^,^29^,^30^,^31^,^32^,^33^,^34^,^35^,^36^. Among the included studies for analysis, there were 16 studies recruited Asian patients while 2 studies recruited Caucasian patients. Five studies collected and measured the peripheral blood sample, and 13 studies used tissue sample. Digestive system cancers were investigated in 10 studies (6 on colorectal cancer, 2 on gastric cancer, 2 on hepatocellular carcinoma) while 2 studies on breast cancer, 2 studies on glioma and 1 studies respectively on cervical cancer, head and neck cancer, laryngeal cancer and non-small cell lung cancer. 15 studies reported mitochondrial DNA copy number as dichotomous variables and 3 studies was divided into three categorical variables. There were 14 studies evaluating overall survival and 6 studies for disease-free survival, and 14 studies assessing high quality and 4 low-quality studies. The characteristics of included studies were presented in Table 1. The detailed extracted data were shown in Supplementary Table S1 and detailed NOS scores of each included study were presented in Supplementary Table S3.

### Data analysis

**Figure 2:**
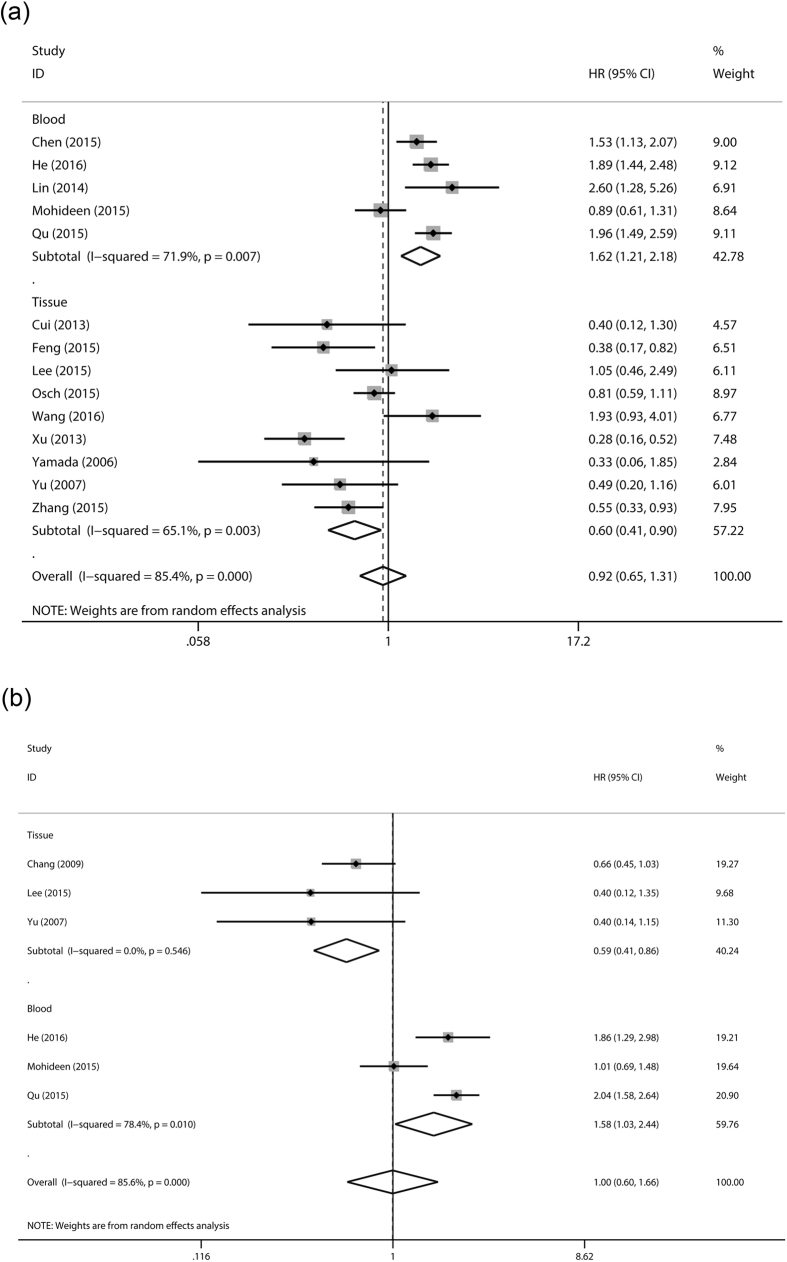
Forest plot of meta-analysis of prognostic role of mtDNA copy number for OS (**a**) or DFS (**b**) stratified by sample.

For overall survival, totally significant heterogeneity (I^2^ = 85.4%) was detected and random-effect model was applied. We didn’t find any association between mitochondrial DNA copy number and overall survival of cancer patients (HR = 0.923, 95% CI: 0.653–1.306, p = 0.652) (Table 2). Significant heterogeneity and negative relationship existed in subgroup stratified by ethnicity, cancer type, case number, analysis method or NOS score. However, in subgroup analysis by sample, the heterogeneity was to some extent decreased and we indicated that high level mitochondrial DNA copy number in peripheral blood predicted a poor cancer prognosis (HR = 1.624, 95% CI: 1.211–2.177, p = 0.001) while high level mitochondrial DNA copy number in tumor tissue was significantly associated with better overall survival in cancer patients (HR = 0.604 95% CI: 0.406–0.899, p = 0.013) (Fig. 2). We further performed detailed stratified analysis in peripheral blood and tissue group (Table 3). We found the significant association between peripheral blood mtDNA copy number and cancer prognosis in Asians (HR = 1.834, 95% CI: 1.564–2.150, p < 0.001), multivariate analysis (HR = 1.532, 95% CI: 1.122–2.092, p = 0.006), studies with larger sample size (HR = 1.532, 95% CI: 1.122–2.092, p = 0.007) and high-quality (HR = 1.532, 95% CI: 1.122–2.092, p = 0.007). The role of mtDNA copy number in tumor tissue on cancer survival also was proved in Asians (HR = 0.567, 95% CI: 0.350–0.918, p = 0.021), multivariate analysis (HR = 0.402, 95% CI: 0.266–0.607, p < 0.001) and high-quality studies (HR = 0.575, 95% CI = 0.356–0.928, p = 0.023).

For disease-free survival, totally significant heterogeneity (I^2^ = 85.6%) also existed and no significant association was detected (HR = 0.997, 95% CI: 0.599–1.659, p = 0.99). In subgroup analysis, we indicated high level mtDNA copy number in tumor tissue predicted better survival (HR = 0.593, 95% CI: 0.411–0.857, p = 0.005) while opposite results were detected in blood (HR = 1.582, 95% CI: 1.026–2.439, p = 0.038) (Table 2, Fig. 2). In more detailed subgroup analysis, tissue mtDNA copy number was also associated with disease-free survival in digestive system cancers (HR = 0.626, 95% CI: 0.423–0.927, p = 0.019). Furthermore, peripheral blood mtDNA copy number was detected to be associated poorer disease-free survival in Asians (HR = 1.989, 95% CI: 1.598–2.476, p < 0.001) (Table 3).

Three studies^25^,^27^,^35^ reported mtDNA copy number as three categorical variables and all used middle level of mtDNA copy number as reference. All three studies recruited Asians and measured copy number in tissue. Cancer type included breast cancer, laryngeal cancer and glioma. However, we didn’t suggested any prognostic role of mtDNA copy number on overall survival between either low level and middle level groups (HR = 1.456, 95% CI: 0.556–3.811, p = 0.444) or high level and middle level groups (HR = 1.654, 95% CI: 0.875–3.124, p = 0.121) (Table 2).

### Publication bias and sensitivity analysis

**Figure 3:**
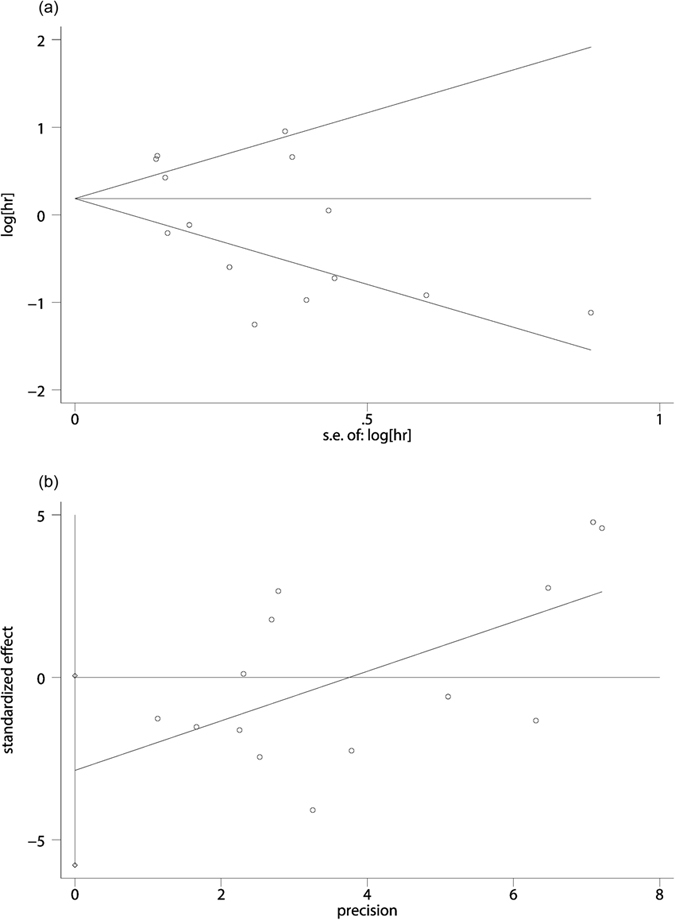
Begg’s funnel plot (**a**) and Egger’s linear regression tests (**b**) for publication bias.

We further investigated the publication bias of our study. We didn’t detected any publication bias in either begg’s (Pr > |z| = 0.228) or egger’s (P > |t| = 0.053) test and suggested the reliability in our meta-analysis (Fig. 3). After dropping each included study, no significant change of our results was found and further proved the stability of our meta-analysis.

## Discussion

Recently, the assessments of prognostic factors of cellular and molecular characteristics are mostly based on one specific-type cancer. Despite the highly heterogeneity of various types of cancers, some clinical denominators like the level of mtDNA copy number is unfolding. To the best of our knowledge, the current meta-analysis was the first comprehensive literature evaluating the role of mtDNA copy number in tissue or serum as a cancer prognostic factor. Hence, we identified eighteen studies including 3961 cases with different cancer types and demonstrated a consistent association between high mtDNA copy number in tissue sample and prognosis of cancer, while in peripheral blood the analysis showed a significant inverse correlation between them. The intriguing finding didn’t appear in the overall analysis probably owing to the evident heterogeneity in these studies. However, in the subgroup analysis stratified by sample, the result implied mtDNA copy number the potential correlation with OS and DFS prognosis.

Given the essential role mitochondria played in cellular functions and energy producing^3^,^4^, many factors causing mitochondrial dysfunction might be not able to maintain the hemostasis of the internal environment, which was postulated to be the fundamental cause of various diseases including cancer^37^,^38^. Among these factors, the qualitative and quantitative changes in nuclear or mitochondrial DNA have been frequently reported in human cancers^26^,^27^,^28^,^29^,^30^,^31^, and the interplay between them may partly participate in the tumor-related process^39^. Previous studies have reported reduced levels of mtDNA transcripts and increased mtDNA copy number in brain and lung with ageing^40^,^41^. An increasing amount of evidence has shown that high mtDNA copy number can be the indicator of ROS-mediated oxidative stress and may be associated with carcinogenesis, while lower mtDNA copy number could cause a deficiency in oxidative phosphorylation resulting enhanced generation of ATP by glycolysis, which often implicated cancer development^42^,^43^. As a promising target for solid cancer, mtDNA copy number attracts the attention of researchers.

One systematic meta-analysis has suggested that the elevated mtDNA copy number was associated with a higher risk for lymphoma but a lower risk for skeleton cancer^15^. Similarly, the correlation between high mtDNA copy number and cancer prognosis was insignificant in the overall evaluation and showed relative large heterogeneity both in the overall survival and disease-free survival analyses. One previous study documented the considerable discrepancy between the mtDNA levels in peripheral blood and tumor tissue extracted from the same patients and suggested the moderate irrelevance of them^44^. Our results further expounded that low mtDNA copy number extracted from tumor tissue was strongly associated with poor survival but the result was opposite in the blood sample. A possible explanation for the low mtDNA copy number of tumor tissue predicting worse outcome was related to the hypoxic condition surrounding the cancer cells. With the development of cancer cell, hypoxia inducible factor (HIF) related to aggravating hypoxia inhibited mitochondrial biogenesis or disrupts mitochondria by mitophagy^45^,^46^, bringing about the lower mitochondrial activity which was regarded as an advantage for cancer progression because of the lower oxidative stress levels^47^. Another explanation was lower mtDNA copy number lead to insufficient oxidative phosphorylation and greater generation of ATP by glycolysis^48^. There was also a possibility that the biosynthesis of mtDNA copy number was unable to catch up with the proliferation of tumors, and poorly differentiated tumors tend to grow and spread at a higher rate than averagely differentiated or well-differentiated tumors^49^. On the contrary, the increasing of mtDNA copy number was generally thought to be the compensatory response to the damaged mitochondrial activity^50^,^51^. These hypotheses were in concordance with one recent study, which seems to follow the inverse U-shape curve of HR with the increasing of copy number to some extent^16^. On account of the mutual interaction between nuclear and mitochondrial genes, the mutation of tumor suppressor genes, oncogene and somatic mtDNA may also be responsible for the abnormal biosynthesis of mtDNA copy number. For example, p53 enhanced mtDNA replication function by interacting with mtDNA polymerase γ and mtDNA^52^. Thus, p53 mutation was associated with decreased mtDNA copy number^26^. In addition, as D-loop containing the transcriptional promoters and the leading strand of the origin of replication of mitochondria, the alteration of D-loop region could also change the mtDNA biogenesis^53^. However, there were contradictory conclusions independent of our included studies as far as the influential factors of mtDNA copy number^34^,^54^. Therefore, future studies need to validate the corresponding mutated genes at specific stages and their separate influence on mtDNA copy number.

It is interesting to find an opposite relation between mtDNA serum level and prognosis comparing to that in tissue. Previous studies have demonstrated the interaction between various immune cells giving rise to the change of the immunological environment contributes to the underlying association. It has been reported that patients with high leukocyte mtDNA copy number have had increased Tregs and decreased NK-cells in peripheral blood, and Tregs were considered as inhibiting anticancer immunity while NK cells served as immunosurveillant, suggesting the patients with high mtDNA copy number awere in the immunosuppressive state^21^,^55^. Moreover, the previous study also showed patients with high plasma mtDNA copy number held lower plasma TNF-α and IFN-ɣ but higher TGF-β1 concentrations^21^. TNF-α enhanced the functions of NK cell but suppressed the Tregs, whereas the TGF-β1 promoted the proliferation and differentiation of Tregs and inhibited the expansion and functions of helper T cells and killer cells. In addition, mitochondrial ROS, generally increased with mtDNA copy number, was an important inducer of TGF-β1^42^,^56^,^57^. Mitochondrial ROS also played an important role in the immune functions of T cells, such as activation and differentiation of naive T cells and apoptosis of activated T cells, and ROS also induced the dysfunction and apoptosis of NK cells^58^,^59^.

Some limitations in the study should be acknowledged. One of the most important limitation in conducting the meta-analysis was various cut-off values applied for mtDNA copy number levels. Most studies chosen the median ratio of mtDNA content of the tumor tissue to that of the pathologically normal tissue surrounding the tumors from the same individual (T/N) as the cutoff value and some compared to the normal mtDNA copy number directly. Other important metrics, such as PCR primers, internal reference gene and treatments during the follow-up months might varied among studies and lack of a standard consensus in some clinical information were unavailable to be considered in our study. In addition, lots of studies recruited specific populations such as Asian and no studies focusing on African patients. And more, we didn’t detect any association in pooled results of three categorical variables. These might be caused by the limited number of included studies presented mtDNA copy number as three categorical variables and the significance between three categorical variables might be smaller than that between dichotomous variables. Furthermore, while our study suggested the interesting opposite outcomes, we should implement more clinical researches to refine the output combining with other parts, and more studies investigating the exact mechanism of different role of mitochondrial DNA copy number in blood or tissue were warranted in future.

In conclusion, elevated mitochondrial DNA copy number in peripheral blood was associated with a poor prognosis of cancer patients while elevated mitochondrial DNA copy number in tumor tissue predicted as a better outcome, especially in Asians. The widespread impact of mtDNA copy number level on clinical parameters including the prognosis of cancer patients seems counterintuitive with regard to the diversity of cancer types, but the conclusions we draw from pooled analyses can have longer term repercussions in the scientific research on mtDNA copy number, Subsequently applied to guide the development of surgical treatment and the choice of postoperative management.

## Method

### Search strategy

A systemic search was performed in PubMed, EmBase, Web of Science databases update to May 23^th^, 2016. The following keywords were used as search terms: “cancer”, “carcinoma”, “neoplasm”, “neoplasia”, “myeloma”, “lymphoma”, “leukemia”, “leiomyoma”, “survival”, “outcome”, “prognosis”, “prognostic” “mitochondrial DNA”, “mtNDA”, “copy number”, “content”. There was no language restriction in our study. The reference of related studies were reviewed manually for potential eligible studies.

### Inclusion and exclusion criteria

Studies met the following criteria were included in our study: (1) the study investigated the association between peripheral or tissue mitochondrial DNA copy number and cancer prognosis; (2) the hazard ratio (HR) with 95% confidence interval (95% CI) were applied to access the strength of mitochondrial DNA copy number and cancer prognosis; (3) HR and 95% CI could be extracted in univariate or multivariate analysis of Cox hazard model, or could be estimated by Parmar’s method^23^. Studies were excluded if they were: (1) reference abstracts, conference report, animal studies, reviews, or meta-analysis; (2) data were not available in extraction or estimation of HR and 95% CI; (3) duplicated publications. If studies with overlapped cases were met, the study with the largest sample size was included.

### Data extraction

Two reviewers extracted the data independently with a standard extraction table. If any disagreement was met, a discussion was conducted for a final conclusion. The following information were extracted: first author’s name, publication year, ethnicity of patients, cancer type, source of sample, number of patients, follow-up period, HR with 95% CI of prognosis.

### Quality assessment

A quality assessment of the included studies was evaluated by two independent investigators on the basis of the Newcastle–Ottawa quality assessment scale (NOS), and conflicting judgments were discussed with a third reviewer until a consensus was reached^24^. NOS is a 9-point scoring system and studies with an NOS score >6 were considered high-quality studies (The Newcastle–Ottawa quality assessment scale was presented in Supplementary Table S2).

### Statistical analysis

Heterogeneity test was performed by Q test and I^2^ test. Acceptable heterogeneity was indicated when Q test p value >0.05 or the result of I^2^ test less than 50% and fixed-effect model was used for further data synthesis. Otherwise, significant heterogeneity was suggested to exist and random-effect model was applied. HR with 95% CI were used to evaluate the role of mitochondrial DNA copy number on cancer prognosis. For more precise results, studies divided mitochondrial DNA copy number as dichotomous or three categorical variables were analyzed separately. Statistical significance was indicated when p value less than 0.5 in data synthesis. If both univariate and multivariate analysis data were available in study, we only included outcomes conducted by multivariate analysis. Furthermore, we performed subgroup analysis stratified by ethnicity, cancer type, sample, case number and survival analysis. We supposed the sample might be the great influential factor and thus more detailed stratified analyses were conducted in sample subgroups. We also evaluated publication bias by begg’s and egger’s test. Sensitivity analysis by leave-one out approach was conducted to detect the stability of outcomes. All analyses were conducted by Stata 14.0 (STATA Corporation, College Station, TX, USA).

## Additional Information

**How to cite this article**: Chen, N. *et al*. Elevated Mitochondrial DNA Copy Number in Peripheral Blood and Tissue Predict the Opposite Outcome of Cancer: A Meta-Analysis. *Sci. Rep*. **6**, 37404; doi: 10.1038/srep37404 (2016).

**Publisher's note**: Springer Nature remains neutral with regard to jurisdictional claims in published maps and institutional affiliations.

## Supplementary Material

Supplementary Information

## Figures and Tables

**Table 1:** The baseline characteristics of included studies.

Author, year	Ethnicity	Cancer type	Sample	N^a^	Male	Female	Survival	Analysis	mtDNA gene	nDNA gene	NOS score
Bai^25^	Asian	Breast cancer	Tissue	148	0	148	OS	MV	tRNA-Leu (UUR)	B2M	4
Chang^26^	Asian	Colorectal cancer	Tissue	194	134	60	DFS	MV	MT-CYB	RNase P gene	9
Chen^20^	Asian	Glioma	Blood	336	189	147	OS/PFS	MV	MT-ND1	HGB	7
Cui^18^	Asian	Colorectal cancer	Tissue	60	30	30	OS	UV	MT-ND1	β-actin	9
Dang^27^	Asian	Laryngeal cancer	Tissue	204	197	7	OS	MV	MT-ND2	β-actin	9
Feng, 2015	Asian	Cervical cancer	Tissue	122^b^	NA	NA	OS	MV	NC_012920 region	B2M	8
He^21^	Asian	HCC	Blood	618	544	74	OS/DFS	MV	MT-ND1	HGB	9
Lee^22^	Asian	Gastric cancer	Tissue	109	82	27	OS/DFS	UV	MT-COX1	β-actin	6
Lin^28^	Asian	Head and neck cancer	Blood	75	75	0	OS	UV	MT-tRNA^leu^	18 S	6
Mohideen^17^	Caucasian	Colorectal cancer	Blood	273^c^	160	116	OS/DFS	MV	MT-ND2	FASLG	8
Osch^16^	Caucasian	Colorectal cancer	Tissue	655^c^	372	306	OS	UV	D-loop	B2M	8
Qu^29^	Asian	Colorectal cancer	Blood	598	328	270	OS/DFS	MV/UV	MT-ND1	HGB	8
Tu, 2015	Caucasian	Prostate cancer	Blood	1266	1266	0	PFS	MV	MT-ND1	HGB	7
Wang^30^	Asian	Colorectal cancer	Tissue	124^c^	72	90	OS	UV	MT-ND1	β-actin	7
Weerts^31^	Caucasian	Breast cancer	Tissue	186^b^	NA	NA	DMFS	MV	MT-TL1	HMBS	7
Xu^32^	Asian	NSCLC	Tissue	128	95	33	OS	MV	NC_012920 region	B2M	8
Yamada^33^	Asian	HCC	Tissue	31	27	4	OS	UV	mtDNA	β-actin	8
Yu^34^	Asian	Breast cancer	Tissue	59	0	59	OS/DFS	UV	D-loop	β-actin	6
Zhang^35^	Asian	Gastric cancer	Tissue	103	84	19	OS	MV	MT-ND1	β-actin	8
Zhang^36^	Asian	Glioma	Tissue	124	68	56	OS	MV	MT-ND1	β-actin	8

^a^Number of included patients; ^b^Unknown information of gender; ^c^existing missing data.

NOS: Newcastle-Ottawa Quality Assessment Scale; OS: overall survival; DFS: disease-free survival; PFS: progression-free survival; DMFS: distant metastasis-free survival; MV: multivariate analysis; UV: univariate analysis; NSCLC: non-small cell lung cancer; HCC: hepatocellular carcinoma.

**Table 2:** The pooled data on survival of meta-analysis.

Variables	N^a^	Case^b^	Pooled data		Heterogeneity	
			HR (95% CI)	P	*I*^*2*^	*Ph*
Dichotomous variables (High level vs. Low level)						
OS						
Overall	14	3312	0.923 (0.653, 1.306)	0.652	85.40%	<0.001
By ethnicity						
Asian	11	2380	0.928 (0.614, 1.402)	0.722	85.80%	<0.001
Caucasian	2	928	0.841 (0.660, 1.071)	0.159	0%	0.709
By cancer						
Digestive system	7	2468	1.158 (0.789, 1.700)	0.453	80.40%	<0.001
Other	6	844	0.709 (0.352, 1.427)	0.335	88.80%	<0.001
By case number						
>200	5	2604	1.340 (0.934, 1.921)	0.112	85.70%	<0.001
<200	8	708	0.679 (0.388, 1.186)	0.173	77.60%	<0.001
By analysis method						
MV	7	2199	0.899 (0.556, 1.455)	0.666	90.80%	<0.001
UV	6	1113	0.955 (0.573, 1.590)	0.858	67.40%	0.005
By NOS score						
High quality	10	3069	0.878 (0.596, 1.292)	0.508	87.50%	<0.001
Low quality	3	243	1.127 (0.423, 2.999)	0.811	77.40%	0.012
By sample						
Blood	5	1900	**1**.**624** (**1**.**211**, **2**.**177**)	**0**.**001**	71.90%	0.007
Tissue	9	1412	**0**.**604** (**0**.**406**, **0**.**899**)	**0**.**013**	65.10%	0.003
DFS						
Overall	6	1850	0.997 (0.599, 1.659)	0.99	85.60%	<0.001
By ethnicity						
Asian	5	1578	0.964 (0.508, 1.828)	0.909	87.50%	<0.001
Caucasian	1	272	1.010 (0.690, 1.479)	0.959	—	—
By cancer						
Digestive system	5	1791	1.127 (0.672, 1.891)	0.65	86.50%	<0.001
Other	1	59	0.399 (0.138, 1.151)	0.089	—	—
By case number						
>200	3	1488	1.582 (1.026, 2.439)	0.038	78.40%	0.01
<200	3	362	0.593 (0.411, 0.857)	0.005	0%	0.546
By analysis method						
MV	3	1084	1.073 (0.605, 1.902)	0.809	83.40%	0.002
UV	3	766	0.753 (0.204, 2.789)	0.672	86.20%	0.001
By NOS score						
High quality	4	1682	1.274 (0.754, 2.153)	0.365	88.20%	<0.001
Blood quality	2	168	0.398 (0.178, 0.887)	0.024	0.00%	0.993
By sample						
Blood	3	1488	**1**.**582** (**1**.**026**, **2**.**439**)	**0**.**038**	78.40%	0.01
Tissue	3	362	**0**.**593** (**0**.**411**, **0**.**857**)	**0**.**005**	0%	0.546
Three categorical variables						
Low level vs. middle level	3	NA	1.456 (0.556, 3.811)	0.444	71.90%	0.029
High level vs. middle level	3	NA	1.654 (0.875, 3.124)	0.121	38.70%	0.195

^a^Numbers of studies included in the meta-analysis; ^b^Number of included patients.

NOS: Newcastle-Ottawa Quality Assessment Scale; OS: overall survival; DFS: disease-free survival; MV: multivariate analysis; UV: univariate analysis; HR: hazard ratio; 95% CI: confidence interval; P: p value of pooled HR; I^2^: value of Higgins I-squared statistics; Ph: p value of Heterogeneity test; NA: not available.

**Table 3:** The pooled data on survival of detailed analyses in blood or tissue subgroup.

Variables	N^a^	Case^b^	Pooled data		Heterogeneity	
			HR (95% CI)	P	*I*^*2*^	*Ph*
OS for tissue group	9	1412	0.604 (0.406, 0.899)	0.013	65.10%	0.003
By ethnicity						
Asian	8	757	0.567 (0.350, 0.918)	0.021	64.70%	0.006
Caucasian	1	655	0.810 (0.594, 1.105)	0.184	—	—
By cancer						
Digestive system	5	979	0.890 (0.540, 1.467)	0.649	49%	0.095
other	4	433	0.418 (0.302, 0.578)	<0.001	0%	0.422
By analysis						
MV	3	374	0.402 (0.266, 0.607)	<0.001	25.30%	0.252
UV	6	1038	0.811 (0.518, 1.269)	0.359	47.60%	0.089
By case number						
>200	1	655	0.810 (0.594, 1.105)	0.810	—	—
<200	8	757	0.567 (0.350, 0.918)	0.021	64.70%	0.006
By NOS score						
High quality	7	1244	0.575 (0.356, 0.928)	0.023	71.80%	0.002
Low quality	2	168	0.718 (0.337, 1.528)	0.39	35.30%	0.214
OS for blood group	5	1900	1.624 (1.211, 2.177)	0.001	71.90%	0.007
By ethnicity						
Asian	4	1627	1.834 (1.564, 2.150)	<0.001	0	0.46
Caucasian	1	273	0.890 (0.606, 1.307)	0.552	—	—
By cancer						
Digestive system	3	1489	1.519 (0.978, 2.359)	0.063	83.80%	0.002
Other	2	411	1,805 (1.116, 2.919)	0.016	45.40%	0.176
By analysis method						
MV	4	1825	1.532 (1.122, 2.092)	0.007	76.10%	0.006
UV	1	75	2.598 (1.284, 5.256)	0.008	—	—
By case number						
>200	4	1825	1.532 (1.122, 2.092)	0.007	76.10%	0.006
<200	1	75	2.598 (1.284, 5.256)	0.008	—	—
By NOS score						
High quality	4	1825	1.532 (1.122, 2.092)	0.007	76.10%	0.006
Low quality	1	75	2.598 (1.284, 5.256)	0.008	—	—
DFS for tissue group	3	362	0.593 (0.411, 0.857)	0.005	0%	0.546
By cancer						
Digestive system	2	303	0.626 (0.423, 0.927)	0.019	0%	0.44
other	1	59	0.399 (0.138, 1.151)	0.089	—	—
By analysis method						
MV	1	194	0.660 (0.436, 0.999)	0.049	—	—
UV	2	168	0.398 (0.178, 0.887)	0.024	0%	0.993
By NOS score						
High quality	1	194	0.660 (0.436, 0.999)	0.049	—	—
Low quality	2	168	0.398 (0.178, 0.887)	0.024	0%	0.993
DFS for blood group	3	1488	1.582 (1.026, 2.439)	0.038	78.40%	0.01
By ethnicity						
Asian	2	1216	1.989 (1.598, 2.476)	<0.001	0%	0.712
Caucasian	1	272	1.010 (0.690, 1.479)	0.959	—	—
By analysis						
MV	2	890	1.362 (0.749, 2.477)	0.311	77.60%	0.035
UV	1	598	2.040 (1.578, 2.637)	<0.001	—	—

^a^Numbers of studies included in the meta-analysis; ^b^Number of included patients.

NOS: Newcastle-Ottawa Quality Assessment Scale; OS: overall survival; DFS: disease-free survival; HR: hazard ratio; MV: multivariate analysis; UV: univariate analysis; 95% CI: confidence interval; P: p value of pooled HR; I^2^: value of Higgins I-squared statistics; Ph: p value of Heterogeneity test.
