# Supplementary material for: Elevated Mitochondrial DNA Copy Number in Peripheral Blood and Tissue Predict the Opposite Outcome of Cancer: A Meta-Analysis
Source: Sci Rep. 2016 Nov 18;6:37404. doi: 10.1038/srep37404 (PMC5114650; doi:10.1038/srep37404)
Supplement: Supplementary Information [file srep37404-s1.pdf]

Elevated Mitochondrial DNA Copy Number in Peripheral Blood and Tissue Predict the Opposite Outcome of Cancer: A Meta-Analysis

Running head: Mitochondrial DNA Copy Number and Cancer Prognosis

Nan Chen[1,*], Shu Wen[1,*], Xiaoru Sun[1,*], Qian Fang[1], Lin Huang[1], Shuai Liu[1], Wanling Li[1], Meng Qiu[1, 2]

[1] West China School of Medicine/West China Hospital, Sichuan University, Chengdu 610041, China.

[2] Department of Medical Oncology, Cancer Center, the State Key Laboratory of Biotherapy, West China Hospital, Sichuan University, No. 37, Guoxue Alley, Chengdu, Sichuan, 610041, China.

Corresponding author: Meng Qiu

Post Code: 610041, PR China.

E-mail: qiumeng33@hotmail.com

Supplementary table S1 The extracted data on survival of included studies

First Author	Year	N[a]	Cut-off value	HR	95%CI	
					ll	ul
**Dichotomous variables**						
**OS**						
Chen	2015	336	T/N[b]	1.53	1.13	2.07
Cui	2013	60	0.72	0.399	0.123	1.298
Feng	2015	122	1758.18/N[c]	0.378	0.174	0.821
He	2016	618	0.98	1.89	1.44	2.48
Lee	2015	109	1.44	1.049	0.455	2.49
Lin	2014	75	17.55/4.84	2.598	1.284	5.256
Mohideen	2015	273	1	0.89	0.606	1.306
Osch	2015	645	215/382	0.81	0.594	1.106
Qu	2015	598	1.04	1.96	1.49	2.59
Wang	2016	124	N[d]	1.933	0.933	4.006
Xu	2013	128	N[d]	0.285	0.156	0.52
**DFS**						
Chang	2009	194	2/3N[d]	0.66	0.45	1.03
He	2016	618	0.98	1.86	1.29	2.98
Lee	2015	109	1.44	0.396	0.116	1.351
Mohideen	2015	272	1	1.01	0.69	1.48
Qu	2015	598	1.04	2.04	1.58	2.64
Tu	2015	589	2.195	0.641	0.394	1.042
Yu	2007	59	0.793	0.399	0.138	1.149
**PFS**						

Chen	2015	336	NA	1.48	1.08	2.03
Tu	2015	1266	2.195	0.641	0.394	1.042
**DMFS**						
Weerts	2016	204	350/377	0.54	0.3	0.97
**Three categorical variables**						
Bai	2011	148	<0.581	3.37	1.17	9.82
			0.581-0.872	reference		
			>0.872	4.05	1.27	12.87
Dang	2014	204	<0.817	1.78	0.93	3.42
			0.817-1.102	reference		
			>1.102	1.4	0.84	2.35
Zhang	2013	103	<0.806	0.52	0.2	1.38
			0.806-1.194	reference		
			>1.194	1.07	0.37	3.07

[a] N:number of included patients

[b]T: mtDNA content of the tumor tissue; N: mtDNA content of the pathologically normal tissue surrounding the tumor tissue; T/N ratio: the ratio of mtDNA content of the tumor tissue to that of the pathologically normal tissue surrounding the tumors from the same individual;

[c]N: nuclear DNA (nDNA) quantities in samples

[d]N:the amount of mtDNA content in the normal tissue sample

OS: overall survival; DFS: disease-free survival; PFS: progression free survival; DMFS: distant metastasis free survival; HR: hazard ratio; 95%CI: confidence interval; ll: lower limit; ul: upper limit.

Supplementary table S2 Newcastle - Ottawa quality assessment scale for cohort studies

NEWCASTLE - OTTAWA QUALITY ASSESSMENT SCALE

COHORT STUDIES

Note: A study can be awarded a maximum of one star for each numbered item within the Selection and Outcome categories. A maximum of two stars can be given for Comparability

Selection (Max 4 Stars)

1) Representativeness of the exposed cohort
   a) truly representative of the average _______________ (describe) in the community *
   b) somewhat representative of the average ______________ in the community *
   c) selected group of users eg nurses, volunteers
   d) no description of the derivation of the cohort
2) Selection of the non exposed cohort
   a) drawn from the same community as the exposed cohort *
   b) drawn from a different source *
   c) no description of the derivation of the non exposed cohort
3) Ascertainment of exposure
   a) secure record (eg surgical records) *
   b) structured interview *
   c) written self report
   d) no description
4) Demonstration that outcome of interest was not present at start of study
   a) yes *
   b) no

Comparability (Max 2 Stars)

1) Comparability of cohorts on the basis of the design or analysis
   a) study controls for ______________ (select the most important factor) *
   b) study controls for any additional factor * (This criteria could be modified to indicate specific control for a second important factor.)

Outcome (Max 3 Stars)

1) Assessment of outcome
   a) independent blind assessment *
   b) record linkage *
   c) self report
   d) no description
2) Was follow-up long enough for outcomes to occur
   a) yes (select an adequate follow up period for outcome of interest) *
   b) no
3) Adequacy of follow up of cohorts
   a) complete follow up - all subjects accounted for *
   b) subjects lost to follow up unlikely to introduce bias - small number lost > 20 % (select an adequate follow up, or description provided of those lost) *
   c) follow up rate < 80% and no description of those lost
   d) no statement

Supplementary table S3 The detail information of the NOS scores of the included studies

Study	Year	NEWCASTLE - OTTAWA QUALITY ASSESSMENT SCALE OF COHORT STUDIES								
		Selection				Comparability	Outcome			Total
		Q1	Q2	Q3	Q4	Q1	Q1	Q2	Q3	
Bai	2011	0	1	1	1	0	1	0	0	4
Chang	2009	1	1	1	1	2	1	1	1	9
Chen	2015	1	1	0	1	1	1	1	1	7
Cui	2013	1	1	1	1	2	1	1	1	9
Dang	2014	1	1	1	1	2	1	1	1	9
Feng	2015	1	1	1	1	1	1	1	1	8
He	2016	1	1	1	1	2	1	1	1	9
Lee	2015	1	1	1	0	1	1	1	0	6
Lin	2014	1	1	0	1	1	1	1	0	6
Mohideen	2015	1	1	1	1	1	1	1	1	8
Osch	2015	1	1	0	1	2	1	1	1	8
Qu	2015	1	1	1	1	2	1	1	0	8
Tu	2015	1	1	0	1	1	1	1	1	7
Wang	2016	1	1	1	1	0	1	1	1	7
Weerts	2016	1	1	1	1	0	1	1	1	7
Xu	2013	1	1	0	1	2	1	1	1	8
Yamada	2006	1	1	1	1	2	1	1	0	8
Yu	2007	1	1	1	1	1	1	0	0	6
Zhang	2013	1	1	1	1	2	1	1	0	8
Zhang	2015	1	1	1	1	2	1	1	0	8

Q: question
